# Genome sequences of four bacterial strains isolated from organofluorine enrichment cultures

**DOI:** 10.1128/mra.00532-24

**Published:** 2024-07-16

**Authors:** Jennifer L. Goff, Chris Hahn, Rebecca A. Ingrassia, Chanistha Tiyapun, Gray G. Waldschmidt, Emma R. Smith, Miranda N. Marini, Olga Shevchenko, Julia A. Maresca

**Affiliations:** 1 Department of Chemistry, SUNY College of Environmental Science and Forestry, Syracuse, New York, USA; 2 Department of Civil and Environmental Engineering, University of Delaware, Newark, Delaware, USA; 3 Microbiology Graduate Program, University of Delaware, Newark, Delaware, USA; 4 Sequencing and Genotyping Center, University of Delaware, Newark, Delaware, USA; Montana State University, Bozeman, Montana, USA

**Keywords:** *Flavobacterium*, *Nocardioides*, *Ochrobactrum*, *Sphingomonas*, bioremediation, perfluorooctanoic acid, fluoroacetic acid, PacBio

## Abstract

Because fluorinated organic compounds are broadly used and highly persistent, microbes isolated from wastewater may be able to degrade these contaminants. Here, we report the genome sequences of *Flavobacterium* sp. str. WV_118_3, *Nocardioides* sp. str. WV_118_6, *Ochrobactrum anthropi* str. WV_118_8, and *Sphingomonas* sp. str. VL_57B, isolated from wastewater.

## ANNOUNCEMENT

Organofluorine compounds are persistent environmental contaminants, and microbial biodegradation has been proposed as a remediation strategy (1, 2). Here, we isolated four bacterial strains after enrichment on perfluorooctanoic acid (PFOA) or fluoroacetic acid (MFA).

Samples were collected from aeration basins in the Van Lare (VL_57B) and Wilmington (WV_118) wastewater treatment plants (43.237° N, 77.577° W and 39.735° N, −75.518° W, respectively) in April 2019, inoculated into minimal medium (2) with 2 µM PFOA or MFA, and incubated aerobically with shaking at room temperature for 16 months with periodic 1:100 transfers into fresh medium. Both enrichments were archived in 12% glycerol in March 2020 and revived in July 2021 on the same medium. After one transfer of WV_118 into minimal medium amended with 200 µM MFA and incubation for ~1 month, 20 µL was plated on minimal medium (1.5% agar) amended with 1% yeast extract. Four colony types appeared. Two representatives of each type were grown in Luria-Bertani broth (LB, Fisher Scientific, catalog # DF0446-17-3) for ~2 days, then harvested. One colony type was present in the PFOA-amended culture (strain VL_57B), which was grown in LB at room temperature until stationary phase, as determined using OD_600 nm_. A phenol-chloroform DNA extraction was performed (3). The 16S rRNA gene of each isolate was amplified and sequenced using primers 8F and 1492R (4), then compared to NCBI-NR using BLASTn. Strain VL_57B was a *Sphingomonas* species, and the WV_118 isolates were *Flavobacterium*, *Pimelobacter*, and *Brucella* species. Using the same DNA extracts, the genome of one representative of each genus was sequenced.

Single-molecule real-time (SMRT) libraries were barcoded and prepared using the PacBio SMRTbell Express Template Preparation Kit (v3.0). Samples WV_118–8 and VL_57B were sheared to 12 kb using a Megaruptor3 (Diagenode). DNA fragments >6  kb were selected using BluePippin (Sage Science). The average library fragment size was 12  kb, as measured by a fragment analyzer (Advanced Analytical Technologies, Inc.). Sequencing was completed on a PacBio Sequel IIe single-molecule sequencer in one 8M SMRT Cell with a 30-h movie. Samples were demultiplexed using PacBio SMRT Link (v11). Sequencing statistics are reported in Table 1.

**TABLE 1:** Sequencing and assembly information

Genome sequencing, assembly, and annotation statistics								Individual contig statistics					
Strain	PacBio reads	Read N50	Assembly length (bp)	Assembly N50	CDS (RASTtk)	CDS (PGAP)	Assembly GC (%)	Contigs	Description	GC (%)	Length (bp)	Topology	Coverage
*Flavobacterium* sp. str. WV_118_3	116,093	11,824	3,853,966	3,853,966	3,608	3,459	39.4	Contig_1	Chromosome	39.4	3,853,966	Circular	129×
*Nocardioides* sp. str. WV_118_6	39,208	9,900	5,555,804	5,555,804	5,391	5,359	72.7	Contig_1	Chromosome	72.7	5,555,804	Circular	62×
*Sphingomonas* sp. str. VL_57B	186,251	14,314	4,853,307	4,651,785	4,634	4,554	67.6	Contig_1	Plasmid	64.1	201,522	Circular	138×
								Contig_2	Chromosome	67.8	4,651,785	Circular	101×
*Ochrobactrum anthropi* str. WV_118_8	143,102	15,215	5,622,450	2,935,917	5,792	5,371	56.0	Contig_1	Chromosome 1	56.5	2,935,917	Circular	85×
								Contig_2	Chromosome 2	55.8	2,110,101	Circular	84×
								Contig_3	Plasmid	56.3	95,490	Circular	120×
								Contig_4	Plasmid	49.8	156,001	Circular	88×
								Contig_5	Plasmid	62.1	44,596	Circular	332×
								Contig_6	Plasmid	54.9	227,581	Circular	84×
								Contig_7	Plasmid	57.8	52,764	Circular	225×

Genomes were assembled and annotated using KBase (5). Reads <1,000 bp were removed using Filtlong v0.2.1 (6) (*kb_filtlong v0.0.2*). Filtered reads were assembled and polished—including overlap identification and trimming—with Flye v2.9.2 (7) (*kb_flye v0.2.2*). Genome rotation was not performed. All genomes had estimated completeness >99.6% and contamination <1.5% [CheckM v1.1.2 (*kb_Msuite v1.4.2*)]. Mobile genetic elements were predicted using geNomad v1.7.5 (8) in NMDC-EDGE (9). Genomes were annotated using RASTtk v1.073 (10) (*RAST_SDK v1.9.5*), available in the accompanying KBase narrative (11). Assembled genomes were deposited in GenBank and reannotated using the Prokaryotic Genome Annotation Pipeline v.6.7 (12). Default parameters were used except where noted.

Four genomes with circularized chromosomes were assembled (Table 1). Strain VL_57B had one circularized predicted plasmid, and strain WV_118_8 had two chromosomes and five circularized predicted plasmids (Table 1). The other two genomes had a single chromosome each. GTDB-Tk v1.7.0 (13) implemented in KBase (*kb_gtdbtk v1.0.0*) identified the strains as *Sphingomonas* sp. str. VL_57B, *Flavobacterium* sp. str. WV_118_3, *Nocardioides* sp. str. WV_118_6, and *Ochrobactrum anthropi* sp. str. WV_118_8.

## Data Availability

The raw reads and assemblies are available at NCBI, linked to BioProject no. PRJNA1107212. The SRA accession numbers are SRS21189452 (VL_57B) and SRS21189453, SRS21189454, and SRS21189455, for WV_118_3, WV_118_6, and WV_118_8, respectively. The assembled genomes (chromosomes and plasmids) are available with accession numbers JBDHNC000000000 (VL_57B), CP156060 (WV_118_3), CP156059 (WV_118_6), and JBDHNC000000000 (WV_118_8). The raw reads, assemblies, and RASTtk annotations are also available in the KBase narrative doi.org/10.25982/179975.10/2345704 (11).
